# Complete Genome Sequence of Multiple-Antibiotic-Resistant Streptococcus parauberis Strain SPOF3K, Isolated from Diseased Olive Flounder (Paralichthys olivaceus)

**DOI:** 10.1128/genomeA.00248-18

**Published:** 2018-04-26

**Authors:** Yoonhang Lee, Thanh Luan Nguyen, Ahran Kim, Nameun Kim, Heyong Jin Roh, Hyun-Ja Han, Sung-Hee Jung, Mi-Young Cho, Ho Young Kang, Do-Hyung Kim

**Affiliations:** a Department of Aquatic Life Medicine, Pukyong National University, Busan, Republic of Korea; b Pathology Research Division, National Institute of Fisheries Science, Busan, Republic of Korea; c Department of Microbiology, Pusan National University, Busan, Republic of Korea

## Abstract

Here, we report the complete genome sequence of multiple-antibiotic-resistant Streptococcus parauberis strain SPOF3K, isolated from the kidney of a diseased olive flounder in South Korea in 2013. Sequencing using a PacBio platform yielded a circular chromosome of 2,128,740 bp and a plasmid of 23,538 bp, harboring 2,123 and 24 protein-coding genes, respectively.

## GENOME ANNOUNCEMENT

Streptococcus parauberis is a Gram-positive and alpha-hemolytic bacterial pathogen that causes udder mastitis in dairy cows and epizootics of streptococcosis in fish. In recent years, S. parauberis has become a dominant causative agent of streptococcosis on olive flounder farms in South Korea (1, 2). Previous studies (2, 3) have revealed a high rate of antibiotic resistance in strains of S. parauberis, indicating that it might be difficult to treat this infection using chemotherapy. A Gram-positive cocci strain isolated from the kidney of a diseased olive flounder has been identified as S. parauberis by PCR assay, using species-specific primers (4). Antimicrobial susceptibility tests based on Clinical and Laboratory Standards Institute guidelines (5) have revealed that this strain is resistant to multiple antibiotics, including ampicillin, amoxicillin, doxycycline, oxytetracycline, gentamicin, florfenicol, erythromycin, oxolinic acid, and sulfamethoxazole-trimethoprim.

Genomic DNA was extracted from S. parauberis strain SPOF3K using a Wizard genomic DNA purification kit (Promega, USA). Whole-genome sequencing was performed with PacBio 20K (Pacific Biosciences) single-molecule real-time (SMRT) technology. Raw sequences were assembled using PacBio SMRT Analysis 2.3.0. Sequencing results yielded a total of 101,064 PacBio reads, with a coverage of 703.34×. Two gapless contigs (2,128,740 bp and 23,538 bp) were obtained after sequence assembly. Gene prediction was conducted using tRNAscan-SE (6) for tRNA search, Rfam (7) for rRNA and noncoding RNA search, and Prodigal (8) for coding sequence (CDS) search. Predicted genes were functionally annotated by homology search against Kyoto Encyclopedia of Genes and Genomes (KEGG) (9), SEED (10), Swiss-Prot (11), and eggNOG (12) databases.

Results indicated that the genome harbors a circular 2,128,740-bp chromosome (contig 1), containing 69 tRNAs, 18 rRNA genes, and 2,123 CDSs, with an average G+C content of 35.67%. The genome also contains a plasmid (contig 2) of 23,538 bp, containing 24 CDSs with an average G+C content of 34.62%. A total of 1,951 genes were assigned to functional categories based on Clusters of Orthologous Groups (COG) assignments (13). Gene category analysis showed that genes related to carbohydrate transport and metabolism comprised the greatest percentage (204 genes, 10.46%), followed by genes associated with replication, recombination, and repair (178 genes, 9.12%). Average nucleotide identity (ANI) analysis (14) revealed that this strain shared 98.43 to 99.87% identities with genome sequences of 15 S. parauberis strains and <81% sequence identities with other streptococcal species. Various virulence-related genes, including those responsible for capsular polysaccharide and M protein (15–17), were identified, based on a search against the Virulence Factor Database (18). Several antibiotic resistance-related genes were also identified, based on a search against the Comprehensive Antibiotic Resistance Database (19), including *mfd* (20), *tetS* (21), *emrB* (22), *ant(6)-la* (23), and *pbp2x* (24), indicating that they might be associated with the phenotypic antibiotic susceptibility of this strain. Phylogenic analysis based on 1,299 core genes of 15 S. parauberis strains (including SPOF3K) showed that our strain was grouped with Asian strains but clearly separated from strains obtained from the United States or from cows with udder mastitis. The genome information presented here can be used to improve understanding about the virulence and antibiotic resistance of this strain, as well as the epidemiology of streptococcosis caused by S. parauberis.

### Accession number(s).

The complete genome sequence of this project has been deposited in the NCBI GenBank database under the accession numbers CP025420 (chromosome) and CP025421 (plasmid).
